# RECENT INNOVATIONS IN INTERVENTIONS TO ADDRESS LONELINESS AND SOCIAL ISOLATION AMONG OLDER ADULTS

**DOI:** 10.1093/geroni/igae098.1065

**Published:** 2024-12-31

**Authors:** Ashwin Kotwal, Thomas Cudjoe

**Affiliations:** Chair; Discussant

## Abstract

Recent years have witnessed unprecedented national and international calls to action to enhance social connection and reduce loneliness and social isolation among older adults. These calls include a 2020 National Academy of Sciences, Engineering, and Medicine report, a 2023 advisory by the Office of the Surgeon General, legislation introduced by U.S. Senators Murphy and Representative Flood, and a Commission on Social Connection by the World Health Organization (WHO). However, significant knowledge gaps remain on interventions to address these needs among older adults, particularly for diverse populations, across settings (i.e. health systems, different communities) and locations (nationally and internationally). In this session we will present four interventions that can reduce loneliness and social isolation among older adults. Studies will provide detail on recent innovations in program delivery., with particular attention to adaptations related to the COVID-19 pandemic that have been sustained. The implementation, sustainability, and scaling of interventions will be discussed, including health system-community referral networks, considering recent Medicaid funding opportunities, and new populations served. In addition, the session will include a discussion of differences in approach to interventions in different settings across the United States as well as globally. The session will foster dialogue on ongoing challenges and future directions in intervention development and research. Loneliness and Social Isolation Interest Group Sponsored Symposium

